# Prevalence of Bacteria and Antimicrobial Resistance Genes in Hospital Water and Surfaces

**DOI:** 10.7759/cureus.18738

**Published:** 2021-10-13

**Authors:** Maira Aleem, Abdul R Azeem, Sidra Rahmatullah, Sufyan Vohra, Shumyila Nasir, Saadia Andleeb

**Affiliations:** 1 Biotechnology, Combined Military Hospital (CMH) - Lahore Medical College and Institute of Dentistry, Lahore, PAK; 2 General Medicine, Combined Military Hospital (CMH), Lahore, PAK; 3 Atta-ur-Rahman School of Applied Biosciences, National University of Science and Technology, Islamabad, PAK

**Keywords:** colistin resistance, multi-drug resistant bacteria, beta-lactamases, hospital surfaces, hospital tap water, antibiotic resistance genes, transmission, antimicrobial resistance

## Abstract

Purpose

Antimicrobial resistance (AMR) has become a worldwide environmental and public health problem, causing more than 250,000 deaths per year. Unregulated usage, unsafe hospital practices, and misuse in veterinary contribute to the development of multidrug resistance in various bacteria. Hospital water was hypothesized to be a hotspot for AMR transmission because of (1) increased exposure to antibiotic load, (2) poor drainage and sanitation system, (3) interaction between environmental and clinical microbes. The purpose of the research was to assess the biodiversity and AMR in hospital tap waters.

Methodology

In this study, the microflora of the hospital tap water and hospital surfaces was observed by obtaining water samples from the intensive care unit (ICU), surgical wards, and washrooms. These were processed through membrane filtration and spread on seven different media (Aeromonas Medium, Azide Dextrose Agar, MacConkey Agar, Mannitol Salt Agar, Pseudomonas Cetrimide Agar, Salmonella Shigella Agar, and Thiosulfate Citrate Bile Salts Sucrose Agar). Surface samples were collected from the faucet, basin, and drain and directly spread on the media plates. Isolates were identified using standard bacteriological and biochemical tests.

Kirby-Bauer disk diffusion method was performed using 21 antibiotic disks from 10 different antibiotic classes. They included ampicillin (AMP), amoxicillin (AML), piperacillin-tazobactam (TZP), cefipime (FEP), cefoxitin (FOX), ceftazidime (CAZ), ceftriaxone (CRO), imipenem (IMP), meropenem (MEM), ciprofloxacin (CIP), moxifloxacin (MXF), levofloxacin (LEV), amikacin (AK), gentamicin (CN), tigecycline (TGC), aztreonam (ATM), erythromycin (E), clindamycin (DA), rifampicin (RD), colistin (CT), and chloramphenicol (C). The results were interpreted according to EUCAST guidelines for the antibiogram of the isolates; 38 isolates were selected out of 162 based on different parameters for genotyping and detection of six beta-lactamase genes (*bla*SHV, *bla*TEM, *bla*CTX-M, *bla*OXA, *bla*KPC, *bla*NDM).

Results

Among these 162 isolates, 82 were obtained from water sources and 80 were collected from surfaces (faucet, basin, drain). The isolates included a variety of bacteria including *Aeromonas* spp. (20%), *Klebsiella* spp. (13%), *Staphylococcus aureus* (13%), *Pseudomonas* spp.(10%), *Escherichia coli* (9%), *Vibrio* spp. (8%), *Enterococcus* spp. (6%), *Shigella* spp. (6%), *Salmonella* spp. (4%), *Acinetobacter* spp. (3%), *Staphylococcus epidermitis* (3%), *Streptococci *spp. (2%), *Proteus* spp. (1%), *Citrobacter* spp. (1%), and *Serratia *spp. (1%). A diverse range of microbes were identified including clinically relevant bacteria, which shows that the urban water cycle is already contaminated with multidrug-resistant microflora of the hospital settings. Macrolide and lincosamide showed the highest resistance followed by penicillin, monobactam, and cephalosporins. *bla*SHV and *bla*TEM were prevalent in samples. *bla*NDM was also found which manifests as a real threat since it causes resistance against carbapenems and colistin, antibiotics reserved as a last resort against infections.

Conclusions

This study presented the ground reality of antibiotic resistance in Pakistan and how its subsequent spread poses a great threat to the strides made in the field of medicine and public health. Strict regulations regarding antibiotic usage, hospital effluent, and urban water sanitation must be imposed to curb the devastating effects of this increasing phenomenon.

## Introduction

Antimicrobial resistance (AMR) has become a worldwide environmental and public health issue resulting in more than 700,000 deaths per year [1]. Water is considered to be the most important vehicle for the dissemination of antibiotic resistance in the environment due to its interaction in every compartment and its linkage with the human-associated microbiota. This is especially true for low-income countries where poor hygiene and sanitation practices further aggravate the problem [2]. The major intersection follows two routes, which come together with a full circle. The first one being the discharge of resistant bacteria in the environment through different sources and the second one being the presence of such bacteria in our urban water system which is consumed by the public.

The most significant amount of antibiotic residues and resistant bacteria are injected into the aquatic ecosystem through hospital water [3]. Hospitals in Pakistan have been estimated to produce 25,000 tons of waste each year containing about 1.4 µg/L to 236.6 µg/L of antibiotic residues, which are left untreated and added to surface water as such [4]. Untreated hospital effluents, entering into the municipal sewage pose a greater threat to the community where mixing of sewage water with drinking water is a common phenomenon [5].

This water ultimately makes its way back as contaminated tap water in hospitals and exposes the already ill and immunocompromised patients to several bacteria through ingestion [6]. The water used in hospital washbasins and washrooms has been claimed to have caused many nosocomial infections due to easy transmission channels between points of contact where patients are exposed to water while bathing, washing their hands, exposure to medical equipment, and through health workers and medical personnel [7]. The presence of antibiotic resistance bacteria (ARB) in such settings increases the chance of genetic transmission between microbes and results in increasing the load of antibiotic resistance and probable evolution into multidrug resistance bacteria [8].

The presence of antibiotic residues, pollutants, and nutrients in the wastewater serves as a selective pressure for the microbes to develop resistance and spread it via mutation or horizontal gene transfer [9]. Among many genetic determinants, resistance in Gram-negative microbes is mostly attributed to extended-spectrum β-lactamases (ESBL). ESBL enzymes have the capability to hydrolyze almost all beta-lactams which hinders the first-line defense against many infections. In recent times, *bla*CTX-M has become the most prevalent ESBL and together with *bla*SHV and *bla*TEM contribute to resistance against penicillins, oxyimino-cephalosporins, and monobactams [10]. Carbapenems and Cephamycins were the next drugs of choice against ESBL bacteria but the prevalence of *bla*OXA, *bla*KPC*,* and recently discovered *bla*NDM have also rendered these ineffective against resistant organisms.

To date, no study had been done in Pakistan on bacteria present in hospital water and their antibiotic resistance patterns. Keeping this in view, this study was designed to discern the prevailing issue of increased antibiotic resistance and antibiotic-resistant bacteria in the hospital water and surfaces with the aim to study their genes and consequent prevalence to better understand the dissemination of these organisms in the water and hospital environment.

## Materials and methods

Sample collection

Three governmental tertiary care hospitals located in different cities were targeted for the sample collection from both water and surface sources to include and observe a range of microbiota. Intensive care units (ICU), surgical wards, and washrooms were selected to check the prevalence of bacteria and antibiotic resistance. The water samples were collected in a 50 ml falcon tube after letting the tap run for two to three minutes to flush out the cold water. The tubes were then, sealed with the film to prevent contamination.

Swab samples were obtained through sterile swab sticks at three different sites: faucet, basin, and drain to study the bacterial inflow from the water system, the bacterial retention at sinks, and ultimate outflow to sewerage through the drainage system. After scrubbing on the respective surfaces, swabs were suspended in the transport medium and capped tightly to prevent contamination. All the samples were put in the ice container and processed within six hours of sampling with Karachi samples taking a few hours more. The experiments were performed in duplicates.

Sample processing

The water was passed through the membrane filter of 0.45 µm and then placed gently on the nutrient medium with the sterile forceps. This was incubated at 37 °C for 24 hours for the enrichment of microbes. The growth was observed the next day and the bacterial suspension was made in 1 ml sterile saline solution. This was then, spread onto seven different media (Aeromonas Medium, Azide Dextrose Agar, MacConkey Agar, Mannitol Salt Agar, Pseudomonas Cetrimide Agar, Salmonella Shigella Agar, and Thiosulfate Citrate Bile Salts Sucrose Agar) to allow the growth of a multitude of microbes according to the nutritional specifications and conditions. Likewise, the surface samples were directly spread on the mentioned media on the first day of sampling and incubated at 37 °C for 24 hours. Different colonies were picked and identified using standard bacteriological analysis protocols and biochemical tests.

Antibiotic susceptibility assay

This assay was performed using the standard Kirby-Bauer disk diffusion method on Mueller Hinton Agar (MHA) with EUCAST guidelines for the antibiogram of the isolates. The inoculum was prepared by suspending 24 hour fresh colonies in 1 ml of sterile saline solution equating the turbidity to that of 0.5 McFarland and swabbed on the media plate. Twenty-one antibiotics disks were dispensed on the surface namely including ampicillin (AMP), amoxicillin (AML), piperacillin-tazobactam (TZP), cefipime (FEP), cefoxitin (FOX), ceftazidime (CAZ), ceftriaxone (CRO), imipenem (IMP), meropenem (MEM), ciprofloxacin (CIP), moxifloxacin (MXF), levofloxacin (LEV), amikacin (AK), gentamicin (CN), tigecycline (TGC), aztreonam (ATM), erythromycin (E), clindamycin (DA), rifampicin (RD), colistin (CT), and chloramphenicol (C). These agents were chosen on the basis of their importance in treating bacterial infections and for diverse representation of different antimicrobial classes. The plate was incubated at 37 °C for 18-24 hours and examined. The diameters of the zone of inhibition were measured against each antibiotic including the diameter of the disk.

Isolation of genomic DNA

Genomic DNA was extracted using the salting-out method where the fresh overnight liquid cultures are used for the isolation of DNA. Of the 162 isolates, only 38 (Table 1) were chosen for genotyping based on the following criteria: the geographical distribution, the varied sources, and higher antibiotic resistance.

**Table 1:** Selection of isolates: 38 isolates were selected keeping in mind their geographical distribution, their source, and their resistance pattern.

Selected AMR isolates (RWP)			Selected AMR isolates (LH)			Selected AMR isolates (KH)		
Isolate	Source	Probable isolate	Isolate	Source	Probable Isolate	Isolate	Source	Probable Isolate
R1K1	ICU	*Klebsiella* spp	L2K1	ICU	Escherichia coli	K3A1	ICU	*Aeromonas* spp
R2K1	ICU	*Salmonella* spp	L1S2	ICU	*Shigella* spp	K2K1	ICU	Escherichia coli
R3K2	Ward	Escherichia coli	L4M1	Ward	Staphylococcus aureus	K4K1	Ward	Escherichia coli
R4M1	Ward	Staphylococcus epidermidis	L3A1	Ward	*Aeromonas* spp	K5A1	Ward	*Aeromonas* spp
R5A1	Washroom	*Aeromonas* spp	L5K1	Washroom	Escherichia coli	K7K1	Washroom	*Acinetobacter *spp
R6K1	Washroom	*Klebsiella* spp	L5A1	Washroom	*Aeromonas* spp	K7S1	Washroom	*Shigella *spp
A11	R Faucet	*Aeromonas* spp	K42	L Faucet	*Salmonella* spp	K101	K Faucet	*Acinetobacter *spp
K72	R Faucet	*Klebsiella* spp	T41	L Faucet	*Vibrio* spp	S101	K Faucet	*Shigella *spp
S21	R Basin	Escherichia coli	A52	L Basin	*Aeromonas* spp	P111	K Basin	*Pseudomonas* spp
A22	R Basin	*Aeromonas* spp	K51	L Basin	Escherichia coli	D111	K Basin	*Enterococcus* spp
K32	R Drain	Escherichia coli	S62	L Drain	*Salmonella* spp	D121	K Drain	*Enterococcus* spp
A31	R Drain	*Aeromonas* spp	A61	L Drain	*Aeromonas* spp	S121	K Drain	Escherichia coli
						K1S1	Sea	*Klebsiella* spp
						K1P1	Sea	*Pseudomonas* spp

Liquid culture of 1.5 ml was centrifuged at maximum speed for one minute to achieve the pellet cells. This was repeated two to three times to get a thick pellet. The pellet was suspended in 600 µl lysis buffer by soft pipetting after removing the supernatant. This mixture was incubated at 37 °C for one hour.

Six hundred microliter of 5M NaCl was then added to the mixture for protein precipitation. This was vortexed slowly for 15 seconds before putting it in a centrifuge at 10,000 RPM for 10 minutes. Following the step, the upper aqueous layer was carefully transferred to a new tube. This step was repeated until the white protein layer completely disappeared.

For the precipitation of DNA, 2.5 or 3 volumes of absolute chilled ethanol were added to the separated aqueous layer and gently mixed. This was refrigerated at −20 °C for 30 minutes followed by centrifugation at maximum speed with 4 °C temperature for 15 minutes. After discarding the supernatant, the DNA was washed with 1 ml of chilled 70% ethanol, which was centrifuged at the same conditions for two minutes. The supernatant was discarded and the DNA pellet was air-dried at room temperature by inverting the tube on a paper towel. The DNA was then suspended in 600 µl TE Buffer after drying.

PCR for detection of antibiotic-resistance genes

Six β lactam genes were subject to be detected in the selected isolates namely: *bla*SHV*, bla*TEM,* bla*CTX-M*, bla*NDM*, bla*OXA*, *and* bla*KPC*. *The primer sequence and amplicon size are given in Table 2. Each PCR reaction consisted of 1× reaction buffer, 1.2 µl of 50 mM MgCl_2_, 0.5 µl of 0.2 mM dNTPs, 2 µl of 1 mM of each primer, 0.2 µl *Taq* DNA polymerase, and 2 µl of target DNA (final reaction volume of 25 µl). PCR products were electrophoresed through 2% agarose and ethidium bromide staining.

**Table 2:** Amplicon size of detected genes: Six β lactam genes were subject to be detected in the selected isolates namely: SHV, TEM, CTXM, NDM, OXA, and KPC. The primer sequences and amplicon size is given in the table.

Primer	Primer sequence (5’-3’)	Amplicon size
*bla*SHVF	CGCCATTACCATGAGCGATA	86
*bla*SHVR	CGCAAAAAGGCAGTCAATCC	
*bla*TEMF	AAGTTGCAGGACCACTTCTG	202
*bla*TEMR	GCACCTATCTCAGCGATCTG	
*bla*CTX-MF	CGATGTGCAGTACCAGTAA	585
*bla*CTX-MR	TTAGTGACCAGAATCAGCGG	
*bla*NDMF	GGTTTGGCGATCTGGTTTTC	621
*bla*NDMR	CGGAATGGCTCATCACGATC	
*bla*OXAF	GCGTGGTTAAGGATGAACAC	438
*bla*OXAR	CATCAAGTTCAACCCAACCG	
*bla*KPCF	CGTCTAGTTCTGCTGTCTTG	798
*bla*KPCR	CTTGTCATCCTTGTTAGGCG	

## Results

Bacterial diversity

**Figure 1:**
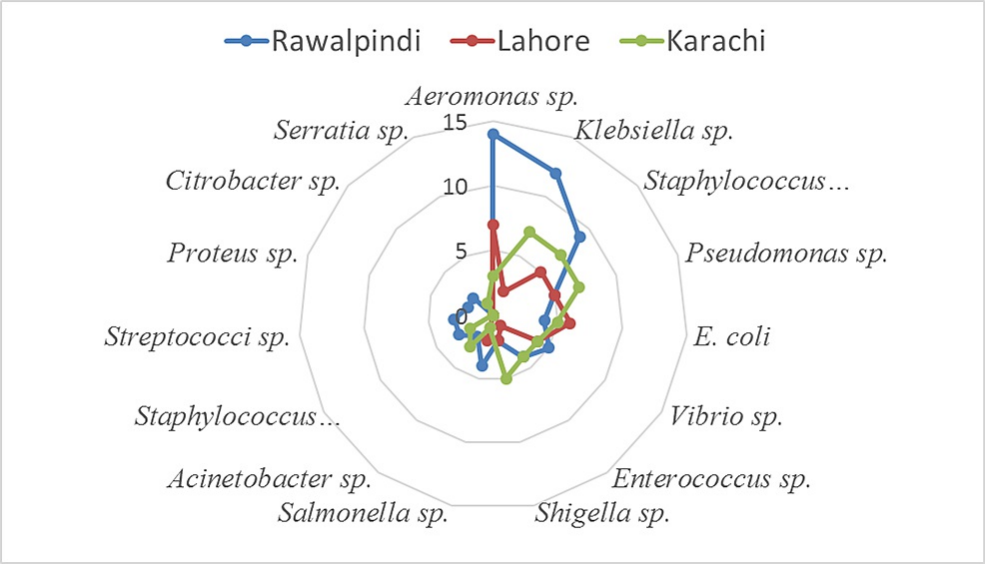
Illustrates the variety of species isolated from three different cities of Pakistan.

**Figure 2:**
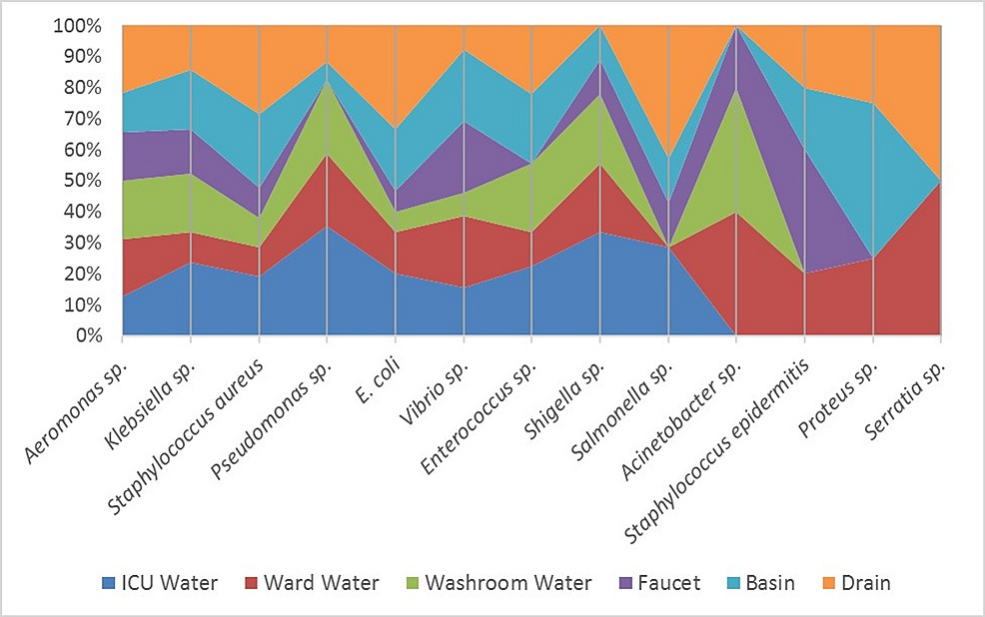
Illustrates the variety of species isolated from three selected water and surface sources each.

A total of 162 isolates were obtained from three major cities of Pakistan Rawalpindi (71), Lahore (57), and Karachi (34) as shown in Figure 1. Among these 162 isolates, 82 were obtained from water sources and 80 were collected from surfaces (faucet, basin, and drain). The isolates included a variety of bacteria including *Aeromonas* spp. (20%), *Klebsiella* spp. (13%), *S. aureus* (13%), *Pseudomonas* spp. (10%), *E. coli* (9%), *Vibrio* spp. (8%), *Enterococcus* spp. (6%), *Shigella* spp. (6%), *Salmonella* spp. (4%), *Acinetobacter* spp. (3%), *S. epidermitis* (3%), *Streptococci *spp. (2%), *Proteus* spp. (1%), *Citrobacter* spp. (1%), and *Serratia *spp. (1%). Among the three surface sources checked, the drain was the most contaminated which suggests that the healthcare workers and patients who wash their hands in the basin are involved in spreading clinical bacteria into the drainage, which ultimately leads to sewage lines. The faucet was the least contaminated source found (Figure 2).

Antibiotic susceptibility

**Figure 3:**
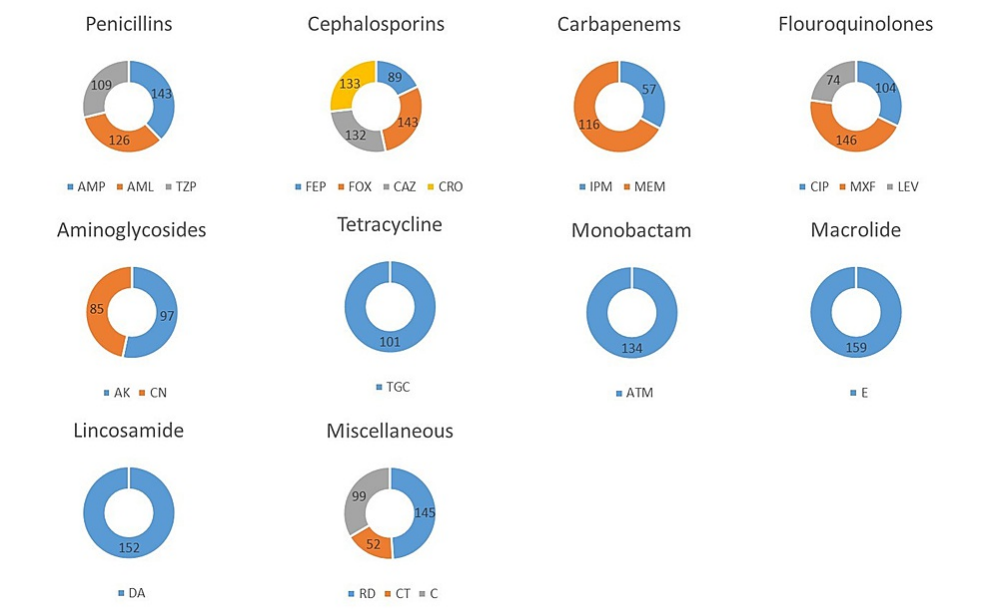
Illustrates the antibiotic resistance of 162 water and surface isolates. Ampicillin (AMP), amoxicillin (AML), piperacillin-tazobactam (TZP), cefipime (FEP), cefoxitin (FOX), ceftazidime (CAZ), ceftriaxone (CRO), imipenem (IMP), meropenem (MEM), ciprofloxacin (CIP), moxifloxacin (MXF), levofloxacin (LEV), amikacin (AK), gentamicin (CN), tigecycline (TGC), aztreonam (ATM), erythromycin (E), clindamycin (DA), rifampicin (RD), colistin (CT), and chloramphenicol (C).

Among the 10 different classes of antibiotics tested, most resistance against macrolides was observed while resistance against fluoroquinolones was the least with levofloxacin being the most effective in this class as shown in Figure 3. Overall, colistin was found to be the most effective antibiotic with 52 (32%) resistance among 162 isolates. Out of the total 162 isolates, about 118 (73%) were extensively drug-resistant (XDR), 28 (17%) were multidrug-resistant (MDR) while only 16 (10%) were susceptible. Among Rawalpindi water isolates, tap water from washrooms contained the maximum number of XDR bacteria followed by tap water from ICUs.

**Figure 4:**
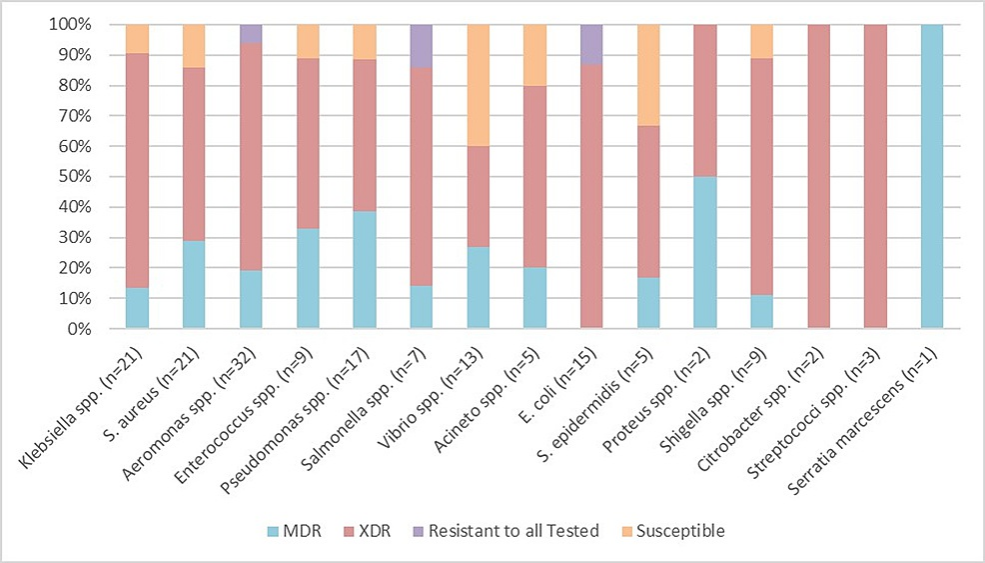
Illustrates the antibiotic resistance in all isolates of bacteria.

Among the surface isolates, drug resistance was more profound in Rawalpindi drain isolates, in which 23% were resistant to all the drugs tested while 92% of the basin surface and 75% of faucet surface isolates were also found to be XDR. Isolates from Lahore had 100% XDR bacteria in ICU tap water and washroom tap waters. Among the Lahore faucet, basin, and drain surface isolates, XDR bacteria were found to be 100%, 67%, and 83%, respectively. Among the tap water samples collected from Karachi, ICU water samples showed 60% XDR bacteria while ward water isolates showed 67% XDR bacteria. Among the faucet, basin, and drain surface isolates, XDR were tested to be 80%, 67%, and 77%, respectively (Figure 4).

All isolates of *Streptococci* and *Citrobacter* were tested to be XDR. *Klebsiella* spp. were found to have 81% XDR isolates and 14% MDR isolates as shown in Figure 4. Similarly, *S. aureus* also had a higher ratio of 1.8 XDR to MDR bacteria. *E. coli*, another common bacteria showed 13% of isolates that were resistant to all the antibiotics tested while the remaining 87% of its isolates were found to be XDR. *Salmonella* spp. showed 14% isolates that were resistant to all the antibiotics tested. *Enterococcus* spp. also had 56% of the isolates classified as XDR. *Aeromonas*, an environmental bacteria and a fish pathogen, had 6% isolates that were resistant to all the antibiotics tested, which is a matter of great concern.

Prevalence of beta-lactam genes

**Figure 5:**
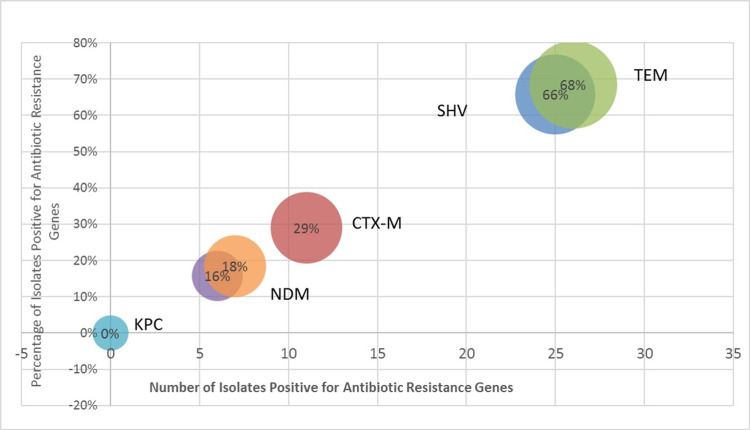
Illustrates the number of genes detected in selected isolates.

**Figure 6:**
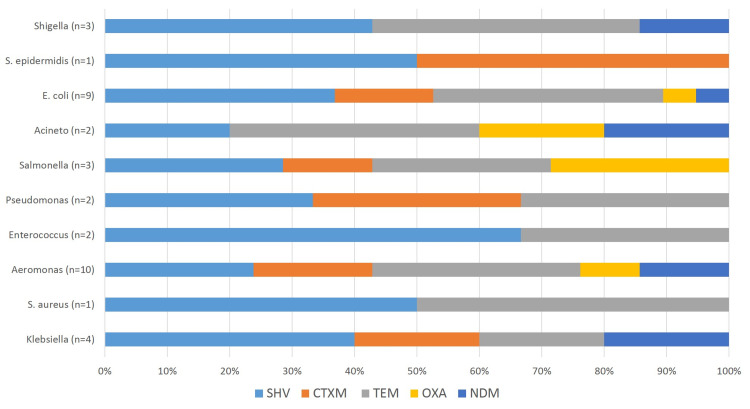
Illustrates the antibiotic resistance genes in bacterial isolates.

The isolates showed a varied presence of the six beta-lactam genes, the most prevalent being *blaTEM* (68%) with* blaSHV* (66%) following closely behind. *blaCTX-M* was found in 29% with 18% *blaNDM* and 16% *blaOXA*. There was no *blaKPC* gene detected. The overall prevalence of genes was detected to be higher in Lahore isolates. The Karachi isolates showed no detection of the *blaCTX-M* gene, however, it had the highest frequency of *blaSHV* (26%). Rawalpindi isolates showed the highest frequency of *blaSHV* (16%) and *blaCTX-M* (16%). Among the species, *Aeromonas* spp. and *E. coli* were found to possess all five genes that were detected while *Acinetobacter* spp., *Salmonella* spp., and *Klebsiella* spp. also harbored four out of five genes. The highest prevalence of *blaNDM* was found in *Acinetobacter* spp. (Figures 5-6).

## Discussion

The hospital environment is an intricate ecosystem that has usually been overlooked as a potential reservoir for bacteria but with the rate of nosocomial infections on the rise; this has become a critical area for the study of microbes. The transmission of pathogens via surface contamination, lack of proper handwashing practices among health care workers, and the water system increased the incidence rate of hospital-acquired infections among the patients [11].

Furthermore, the increase in the antibiotic resistance patterns of the microbes has forced the scientific community to look into the source of such pathogens and their mechanisms of acquired resistance in different environmental compartments. Water bodies were also considered to be a reservoir for the antibiotic resistance, especially because it facilitates the interaction of pathogenic bacteria with the non-pathogenic ones and contribute to the increase in resistance [12].

In the present study, of 162 isolates, 127 were Gram-negative while only 35 isolates were Gram-positive. Similar ratios were found in other studies that observed the bacterial diversity in hospital settings with the prevalence of Gram-negative bacteria [13]. In a study conducted on river waters, 81.8% of *Aeromonas* samples were found to be multidrug-resistant to the most commonly used antibiotics [14]. The results are in coherence with our study where 100% resistance was shown towards ampicillin and more than 80% towards amoxicillin, aminoglycosides, and macrolides. Cephalosporins, which were considered to be suitable drugs [15] against *Aeromonas* also showed more than 80% resistance to ceftriaxone whereas susceptibility to ciprofloxacin has decreased to 44%, concurrent with the study [16].

Similarly, *Klebsiella* species has also shown an increasing trend of resistance against commonly used antibiotics with cefotaxime resistance increased from 75% to 94%, ciprofloxacin went from 64% to 84%, and carbapenems from 2.4% to 52% [17]. The trend in our study is on the higher end of the spectrum with 90% resistance to ciprofloxacin, cefoxitin, and erythromycin, 80% towards ceftriaxone, and 40% towards carbapenems. Carbapenem resistance is owed to recently discovered,* blaNDM* genes and *blaCTXM*, both of which were present in our isolates. This leaves colistin as the only suitable option with 70% susceptibility in *Klebsiella* isolates coherent with a study done in Europe [18].

*Pseudomonas* species was the third most common organism isolated in our study with 90% isolates as multidrug-resistant. More than 80% resistance was shown towards cephalosporins, penicillins, and macrolides coherent with other studies [19]. The least resistance was observed against ciprofloxacin (30%) and colistin (10%) supported by a study done in Karachi [20]. This pattern of resistance is mainly due to the expanded beta-lactam activity of these strains which is contributed to beta-lactamase genes. Our tested isolates were shown to carry extended-spectrum beta-lactamase genes* blaTEM, blaSHV,* and *blaCTXM*. The frequencies of *blaTEM* and *blaSHV* genes in the bacterial isolates were calculated to be 46% and 34%, respectively [21], in another study which is similar to our findings.

A study conducted on water samples from hospital sources found *S. aureus *to be the most predominant organism. Comparatively, it constituted only 13% of total isolates in our study. Different studies have shown *Staphylococcus* to be multidrug-resistant with 100% resistance against amoxicillin, streptomycin, ceftriaxone, and erythromycin, 83% to gentamycin and 78% to cefoxitin and ciprofloxacin [21]. These findings were supported by our study as more than 80% of resistance was shown towards these drugs classes. A shifting trend was observed in our study from a study that reported amikacin and levofloxacin as a susceptible antibiotic against Staphylococcal isolates where resistance has increased to 58% and 52%, respectively, with 96% sensitivity to imipenem as reported in a study from Peshawar [21]. *Staphylococcus epidermidis* also exhibited resistance to macrolides, cephalosporins, and fluoroquinolones as reported in other studies [22]. Researchers have associated multidrug resistance with *blaTEM* genes along with *blaCTXM* and *blaSHV* that were prevalent in our Staphylococcal isolates.

Organisms like *E. coli, Vibrio, Enterococci,* and *Shigella* have been known to cause water-borne diseases like cholera and dysentery. Combined, they made 23% of the total isolates. Similar resistance patterns were observed in isolates of *E. coli* with 100% resistance towards cephalosporins, more than 90% resistance against ampicillin, more than 80% resistance towards macrolides, and 68% against fluoroquinolones. This was also supported by a study that reported 100% resistance towards penicillins, more than 80% towards first and second-generation cephalosporins, and 20% towards fluoroquinolones and macrolides [23]. On contrary, *Vibrio* species showed 90% susceptibility to fluoroquinolones and 80% susceptibility to carbapenems consistent with findings in China [24] but resistance patterns were similar against penicillins, erythromycin, and clindamycin as in other isolates in our study. The resistance in both *Enterococci* and *Shigella* was 100% towards erythromycin and ceftriaxone, followed by 90% against ampicillin and cefoxitin concurrent with other studies [25]. Resistance against ceftriaxone (20%) and ciprofloxacin (12%) in *Shigella* has risen in the past seven years to 68% and 58%, respectively, in our study compared to the study done in Faisalabad. This overall resistance against all these drugs has been conferred to the bacteria’s innate resistance [26]. *blaSHV* and *blaTEM* have been found to confer resistance against many antibiotics and have been the most prevalent in *E. coli, Vibrio, Enterococci,* and *Shigella. E. coli *had the maximum number of resistance genes including *blaCTXM, blaOXA,* and *blaNDM*.

XDR typhi is an extensively drug-resistant strain of *Salmonella typhi* and is resistant to all the antibiotics recommended for typhoid fever except azithromycin and carbapenems. The isolates in our study have exhibited 100% resistance towards ampicillin, ciprofloxacin, aztreonam, clindamycin, and colistin with similar results reported in other studies [27]. *Salmonella* isolates were found to possess *blaSHV, blaTEM, blaCTXM,* and *blaOXA*, all of which explain the resistance pattern shown by the isolates. ESBL pattern of resistance has also been observed in *Acinetobacter* and *Pneumococci* isolates with the highest resistance against penicillins, cephalosporins, and fluoroquinolones consistent with other findings [28]. Recently, several serotypes of *S. pneumoniae* have been isolated which show 100% resistance towards penicillins and cephalosporins with growing resistance towards macrolides and fluoroquinolones. Growing resistance against chloramphenicol has also been recently discovered where 36% resistance was reported against the drug [29], but it contradicts our findings of 100% sensitivity towards it. Proteus species on the other hand has shown 100% resistance towards chloramphenicol as well as penicillins, cephalosporins (all three generations), and macrolides. It has shown susceptibility towards piperacillin-tazobactam [30] but our findings are contrary to these as 100% resistance was shown towards this drug with only susceptibility towards imipenem and amikacin.

## Conclusions

It was concluded in this study that Gram-negative bacteria have more suited features to be able to survive in the environment for longer periods of time, which is why they are of concern especially, in the hospital setting where the patients are more prone to catch an infection. This study suggests that hospital tap water habitat comprises a diverse range of microbes including the ones that have been identified as clinically relevant. The study also shows that the urban water cycle is already contaminated with the microflora of the hospital settings including *Aeromonas, Klebsiella, Pseudomonas, Staphylococcus,* and *Vibrio* species in abundance that are a threat to hospitalized patients, especially immunocompromised ones.

Most resistance was found against macrolides, lincosamides, monobactams, and penicillins. The most resistant pathogens were found to be in ICU compared to other wards because of more frequent antibiotic usage and the presence of immunocompromised patients, which call for stringent policies and infection control programs in the hospitals. Apart from this, many of the organisms in this study were found to be extended-spectrum beta-lactam (ESBL) producers. This manifests as a real threat, especially with *blaNDM* prevalence on the rise, which might result in resistance against carbapenems and colistin, antibiotics reserved as a last line of defense against infections. Public health measures for clean tap water, clean water supply in hospitals along with information sharing and stimulation of research in this field shall contribute towards bridging gaps and a better understanding of this increasing phenomenon. We need more research in this area involving more hospitals and medical setups around the country.
